# Efficient exogenous DNA-free reprogramming with suicide gene vectors

**DOI:** 10.1038/s12276-019-0282-7

**Published:** 2019-07-19

**Authors:** Minhyung Lee, Jeongmin Ha, Ye Seul Son, Hyunjun Ahn, Kwang Bo Jung, Mi-Young Son, Janghwan Kim

**Affiliations:** 1 Stem Cell Convergence Research Center, Korea Research Institute of Bioscience and Biotechnology (KRIBB), Daejeon, 34141 Republic of Korea; 2 Department of Functional Genomics, KRIBB School of Bioscience, Korea University of Science and Technology, Daejeon, 34113 Republic of Korea

**Keywords:** Reprogramming, Transdifferentiation

## Abstract

Reprogramming with episomal vectors is an easy, safe, and cost-effective method to generate exogenous DNA-free (exogene-free) induced pluripotent stem cells (iPSCs). However, the genomic integration of exogenes is observed occasionally. Additionally, the removal of episomal DNA takes more than 70 days in established iPSCs. Here, we inserted the cytosine deaminase (CD) gene from yeast into episomal vectors and used them to reprogram human fibroblasts into iPSCs. These new episomal vectors (CD episomal vectors) were eliminated from the generated iPSCs as early as seven days after 5-fluorocytosine (5-FC) treatment. We also found that cells with the integration of the CD gene perished within two days of 5-FC treatment. In addition, we generated exogene-free induced neural stem cells after one passage by direct reprogramming with CD episomal vectors combined with 5-FC treatment. Conclusively, our novel method allows the rapid and easy isolation of exogene-free reprogrammed cells and can be applied to disease modeling and clinical applications.

## Stem cells: Cleaner generation by selective killing

Using genetic engineering to convert cells into stem cells without leaving unwanted foreign DNA behind is made easier and more effective by a method that allows cells acquiring unwanted DNA to be selectively killed. Janghwan Kim, Mi-Young Son and colleagues at the Korea Research Institute of Bioscience and Biotechnology, Daejeon, included a cell “suicide” gene in the DNA carrier that is used to transfer desired genes into cells. The suicide gene codes for an enzyme that converts an inactive drug into its active form. If undesired DNA from the carrier integrates into the cell DNA, as occasionally occurs, the action of the suicide gene selectively kills the affected cells. The method will improve the supply of stem cells that are used to study disease, test new drugs, and carry out research on correcting genetic diseases.

## Introduction

Induced pluripotent stem cells (iPSCs) are generated by the delivery of reprogramming factors and are used for individualized disease modeling, drug screening, and regenerative medicine^1^. However, because of the limitations of the original method that utilizes viral vectors, the integration of transgenes into the host cell genome is inevitable^2^. The integrated reprogramming factors themselves may cause oncogenic transformation or may change the characteristics of the cells by the insertional inactivation of host genes^3^. Therefore, nonintegrating methods for the generation of exogenous DNA-free iPSCs (EF-iPSCs), such as the use of mRNAs^4^, miRNAs^5^, proteins^6^, episomal vectors^7^, piggyBac vectors^8^, minicircles^9^, antibodies^10^, or the Sendai virus^11^, have been developed. Among these approaches, the use of episomal vectors is relatively cheap and easily applicable to reprogramming multiple cell types, so it has been widely used to generate EF-iPSCs^12–14^. It is generally recognized that episomal vectors are spontaneously removed from cells during cell division^7^. However, cells that are reprogrammed using this method contain the episomal vectors after 10 passages in culture and show relatively frequent integration of the episomal vectors into the genome^15^.

An enzyme encoded by a suicide gene converts a nontoxic substrate, such as a pro-drug, into a toxic product, which subsequently induces apoptosis^16^. For instance, thymidine kinase and cytochrome P450 are suicide genes that convert ganciclovir and cyclophosphamide, respectively, into toxic metabolites^17,18^. Another suicide gene, cytosine deaminase (CD), catalyzes the conversion of the substrate 5-fluorocytosine (5-FC) into 5-fluorouracil (5-FU), which is a lethal drug^19^. The CD/5-FC combination shows greater cytotoxicity than the thymidine kinase/ganciclovir combination^20^. Moreover, 5-FU induces cell death in neighboring cells via what is known as the bystander effect, and the CD gene is expressed in bacteria and fungi but not in humans^21^. For these reasons, gene therapy that introduces the CD gene could be a potential cancer treatment. Indeed, several clinical trials using the CD/5-FC combination have been reported^22–25^. In stem cells, the CD/5-FC combination has been used as a safeguarding system to eliminate oncogenically transformed and/or undifferentiated pluripotent stem cells in regenerative medicinal applications^26^.

Here, we developed a new episomal vector-based reprogramming method employing the CD/5-FC combination for the easy and rapid isolation of EF-iPSCs and EF-induced neural stem cells (iNSCs) from human fibroblasts. We could negatively select cells with an integrated copy of the CD gene and promptly isolate EF-reprogrammed cells within seven days. We propose that our CD episomal vector system offers the easiest and cheapest method for producing safe reprogrammed cells.

## Materials and methods

### Cell culture

The CRL2097 human fibroblast cell line was purchased from the American Type Culture Collection (ATCC, Rockville, MD, USA). CRL2097 cells were cultured in a human fibroblast medium consisting of MEM supplemented with 10% FBS and 1% sodium pyruvate (all purchased from Thermo Fisher Scientific, Waltham, MA, USA). H9 (WA09) cells were purchased from the WiCell Research Institute (Madison, WI, USA), and HUES9 cells were purchased from the Harvard Stem Cell Institute (Cambridge, MA, USA). Human embryonic stem cells (ESCs) and iPSCs were maintained in a pluripotent stem cell (PSC) medium consisting of TeSR-E8 medium (STEMCELL Technologies, Vancouver, Canada) supplemented with 1 mM nicotinamide (Sigma-Aldrich, St. Louis, MO, USA)^27^. For subculturing, all PSCs were dissociated using Accutase (Millipore, Billerica, MA, USA) and then seeded onto plates coated with Geltrex (Thermo Fisher Scientific) in PSC medium containing 10 µM Y-27632 (Tocris, Bristol, England). The medium was replaced with PSC medium 16–24 h after passaging. All PSCs were passaged every 5–7 days. The ESCs used in this study were reviewed and approved by the Public Institutional Review Board designated by Ministry of Health and Welfare (P01-201409-ES-01). For reprogramming purposes, CRL2097 cells were exempted from IRB review (P01-201802-31-001).

### Vector construction

For pCXLE-hOCT4-CD construction, OCT4 was amplified by PCR from the pHAGE2-EF1aL-hSTEMCCA-W-loxP plasmid. The yeast CD gene was amplified by PCR from the genomic DNA of *S. cerevisiae*. The two fragments were ligated to generate one fragment-linked 2A peptide (P2A) sequence by a Gibson Assembly reaction (New England Biolabs, Ipswich, MA, USA), and PCR was performed for amplification of the ligated fragment. This fragment was purified and used for the TOPO cloning reaction (Thermo Fisher Scientific). The reactant was transformed into chemically competent *E. coli* cells. Some *E. coli* colonies were picked and mapped by PCR. The plasmids from the selected clones were used for the LR cloning reaction (Thermo Fisher Scientific) to pCXLE-GW. An additional round of transformation and selection was performed as described above. Finally, the selected clone had its DNA sequence confirmed by sequencing that was carried out by GenoTech (Daejeon, Republic of Korea). For pCXLE-hSK-CD construction, SOX2-P2A-KLF4 was amplified by PCR from the pHAGE2-EF1aL-hSTEMCCA-W-loxP plasmid. The steps used to generate the construct were the same as those used for the pCXLE-hOCT4-CD construct, except for those steps that involved the internal ribosome entry site (IRES) sequence that was used for linking SOX2-P2A-KLF4 and the CD. For pCXLE-hUL-CD construction, all procedures were performed by Enzynomics (Daejeon, Republic of Korea).

### Reprogramming of human fibroblasts to iPSCs

Reprogramming with episomal vectors was performed as previously described^13^. Briefly, 500 ng of episomal vector mixture was electroporated into 100,000 cells with a Neon electroporator (Thermo Fisher Scientific) using a Neon Transfection System 10 µl Kit (Thermo Fisher Scientific) according to the manufacturer’s instructions. The electroporation conditions used in the experiments were 1650 V, 10 ms, and 3 pulses. The transfected cells were seeded onto Geltrex-coated plates and cultured for 5 days in human fibroblast medium. The culture medium was replaced with mTeSR-1 medium (STEMCELL Technologies) containing 1 mM nicotinamide (Sigma-Aldrich), 0.2 mM sodium butyrate (Sigma-Aldrich), 3 µM CHIR99021 (Tocris), 0.5 µM A83-01 (Tocris), and 50 µg/ml 2-phospho-L-ascorbic acid (Sigma-Aldrich), and the cells were cultured for 13–16 days. The resulting colonies were manually picked and maintained in PSC medium.

### Reprogramming of human fibroblasts to iNSCs

Reprogramming into iNSCs was performed using a previously described method^28^ with slight modifications. Briefly, 10 µg of episomal vector mixture was electroporated into 2,000,000 cells using a NEPA21 Super Electroporator (Nepagene, Japan) according to the manufacturer’s instructions. The transfected cells were seeded onto Geltrex-coated plates and cultured for 5 days in human fibroblast medium. The culture medium was replaced with a RepM-Neural medium which contains Advanced DMEM/F12 and Neurobasal medium mixed at a ratio of 1:1 and supplemented with 0.05% AlbuMAX-I, 1 × N2, 1 × B27 minus vitamin A, 2 mM GlutaMAX, 0.11 mM β-mercaptoethanol (all purchased from Thermo Fisher Scientific), and 10 ng/ml human LIF (Peprotech, Rocky Hill, NJ, USA), and the cells were cultured for 13–16 days. During reprogramming, a chemical cocktail containing 0.2 mM NaB, 3 μM CHIR99021, 0.5 μM A83-01, and 50 µg/ml 2-phospho-L-ascorbic acid was added to the medium before use. The resulting colonies were manually picked and maintained in RepM-Neural medium containing 3 μM CHIR99021 and 0.5 μM A83-01.

### Detection of the episomal vectors

The episomal vector copy number was calculated using a previously described method^29^ with slight modifications. The cultured cells were dissociated using Accutase. The cells were then lysed with DirectPCR Lysis Reagent (Viagen, Cedar Park, TX, USA) to extract the total DNA according to the manufacturer’s instructions. The lysates were stored at −20 °C until use in the quantitative PCR analysis. To determine the episomal vector copy number, a standard curve for the F-box 15 (*FBXO15*) gene or the Epstein-Barr nuclear antigen-1 (*EBNA-1*) gene was generated from the pCXLE-hFbx15-cont2 plasmid using qPCR. The Ct values of FBXO15 and EBNA-1 were used to determine the cell number and the episomal vector copy number, respectively, in each qPCR reaction^7^. The number of copies per cell was calculated by dividing the total episomal vector copy number by the cell number.

To perform PCR for the exogene elements in CD-iPSCs in the presence or absence of 5-FC treatment, total DNA was used as a template. For the positive control, a mixture of three CD episomal vectors was used as the template. For the negative control, genomic DNA from CD-iPSC #01 was used as the template. The PCR reactions were performed using a premixed PCR reagent (Bioneer, Daejeon, Republic of Korea) with a C1000 thermal cycler (Bio-Rad). The primer sequences used in this experiment are shown in Supplementary Table 1.

### Cytotoxicity assay

The ESCs were seeded in 96-well plates, and various concentrations of 5-FC or 5-FU were added to the medium for 48 h. The cytotoxicity of 5-FC and 5-FU were measured using the WST-1 cell proliferation assay system (TaKaRa, Japan) according to the manufacturer’s instructions. The assay was repeated at least three times.

### Lentivirus production

Lentiviral particles were produced by cotransfecting the expression vector (8.75 µg), the packaging vector (3.75 µg), and the envelope vector (2.5 µg) into 293T cells using TransIT-2020 Reagent (Mirus, Madison, WI, USA) according to the manufacturer’s instructions. The 293T medium was composed of DMEM (Thermo Fisher Scientific) supplemented with 10% FBS and was replaced 24 h after transfection. The culture supernatant containing lentiviral particles was harvested 48 and 72 h posttransfection. The supernatant was concentrated using ultracentrifugation and then stored at −80 °C.

### Reverse transcription PCR (RT-PCR)

The RT-PCR analysis was performed as previously described^30^ with slight modifications. Total RNA was extracted from cells using an RNeasy Mini Kit with a QiaShredder (Qiagen, Hilden, Germany) and DNase I (Qiagen) and reverse-transcribed using an iScript cDNA synthesis kit (Bio-Rad, Hercules, CA, USA) according to the manufacturer’s instructions. For each reaction, a 1/50 dilution of the cDNA template was used for PCR with a premixed PCR reagent (Bioneer) on a C1000 thermal cycler (Bio-Rad). For the RT negative control, 1 µg of total RNA was used to synthesize cDNAs in the absence of reverse transcriptase; then, primers for the glyceraldehyde 3-phosphate dehydrogenase (*GAPDH*) internal control sequence were used to detect genomic DNA contamination. The primer sequences used in this experiment are shown in Supplementary Table 1.

### Three-germ layer differentiation

The iPSCs were detached using dispase (Thermo Fisher Scientific) for 20 min at 37 °C, harvested and washed with DPBS twice to determine the differentiation potential of iPSCs in vitro. To generate the embryoid bodies (EBs), collected clumps of cells were cultured for one week in EB medium consisting of DMEM/F-12 supplemented with 10% Knockout Serum Replacement, 5% FBS, 1% minimal essential medium-nonessential amino acids (MEM-NEAA), 1% GlutaMAX, 1% penicillin/streptomycin, and 0.1% 2-mercaptoethanol (all purchased from Thermo Fisher Scientific). The EBs were allowed to adhere to Geltrex-coated culture plates and cultured for an additional week. Teratoma formation was assessed as previously described to elucidate the differentiation potential of iPSCs in vitro^31^. Briefly, iPSCs were injected into nonobese diabetic severe combined immunodeficient mice. After three months, the teratomas were removed, fixed overnight with 10% formalin, embedded in paraffin, and sectioned serially. The sections were stained with hematoxylin and eosin (H&E). All animal experiments and care procedures were approved by the Institutional Animal Care and Use Committee of KRIBB (approval KRIBB-AEC-17014).

### Immunocytochemistry

Immunocytochemistry was performed using a previously described method^32^ with slight modifications. Samples were fixed with 4% paraformaldehyde (Electron Microscopy Sciences, Hatfield, PA, USA) and 0.15% picric acid (Sigma-Aldrich) and then blocked and permeabilized with 3% bovine serum albumin (BSA; Thermo Fisher Scientific) and 0.3% Triton X-100 (Sigma-Aldrich) in DPBS for 1 h at room temperature. All samples were incubated with each primary antibody solution overnight at 4 °C. After washing with 0.1% BSA in DPBS, the samples were incubated with Alexa Fluor 488- or Alexa Fluor 594-conjugated secondary antibodies (all purchased from Thermo Fisher Scientific) for 1 h at room temperature. Images were captured using a Zeiss Axio Vert.A1 microscope (Carl Zeiss, Oberkochen, Germany) and a Leica DMI4000B microscope (Leica, Wetzlar, Germany). The antibodies used in this experiment are described in Supplementary Table 2.

### Alkaline phosphatase (AP) staining

A Leukocyte AP Kit (Sigma-Aldrich) was used according to the manufacturer’s instructions. Briefly, samples were fixed with 10% formalin (Sigma-Aldrich) for 30 s. The fixed samples were washed once with 1 × TBST (LPS solution, Daejeon, Republic of Korea) and then incubated with an AP substrate solution for 20 min in the dark. Images were captured using a Zeiss Axio Vert.A1 microscope (Carl Zeiss).

### Karyotype and short tandem repeat (STR) array analysis

The karyotyping of EF-iPSCs and iNSCs was conducted by GenDix (Seoul, Republic of Korea). An STR array analysis was performed as previously described. Briefly, genomic DNA was extracted from EF-iPSCs, EF-iNSCs, and CRL2097 cells using a DNeasy Blood and Tissue Kit (Qiagen) according to the manufacturer’s instructions. The STR array was analyzed by Humanpass (Seoul, Republic of Korea).

### Calcium imaging

Calcium flux analysis was performed as described previously^28^. Spontaneously differentiated neurons were washed and loaded with 5 μM Fluo-4 acetoxymethylester (Fluo-4 AM, Molecular Probes, Eugene, Oregon, USA) for 1 h. The neurons were stimulated with 32.5 mM KCl (Sigma-Aldrich) in isotonic buffer (5 mM KCl, 10 mM HEPES, 140 mM NaCl, 5.5 mM D-Glucose, 2 mM CaCl_2_, and 2 mM MgCl_2_) and imaged using a confocal microscope (IX83, Olympus, Japan). The cells were exposed to wavelengths of 505–530 nm/488 nm (excitation/emission) to obtain the calcium images. The fluorescence intensities were measured in the regions of interest (ROIs) and analyzed with FV 1000 software (Olympus).

## Results

### Use of a suicide gene to select exogene-free reprogrammed cells

Reprogramming with episomal vectors is well known to be the easiest and most economical method of producing integration-free iPSCs. This might contribute to expanding and generalizing the use of the reprogramming technology. However, there are two limitations for episomal vector-based reprogramming. First, genomic integration of the episomal vectors occurs sometimes^11^. Second, the episomal vectors are persistently maintained even after the establishment of iPSCs. Thus, practically, a number of individual colonies need to be selected and passaged for more than 10 weeks iteratively to isolate EF-iPSCs. To avoid this tedious process, we sought to achieve the active removal of exogene-harboring cells and the fast selection of EF-iPSCs.

**Fig. 1:**
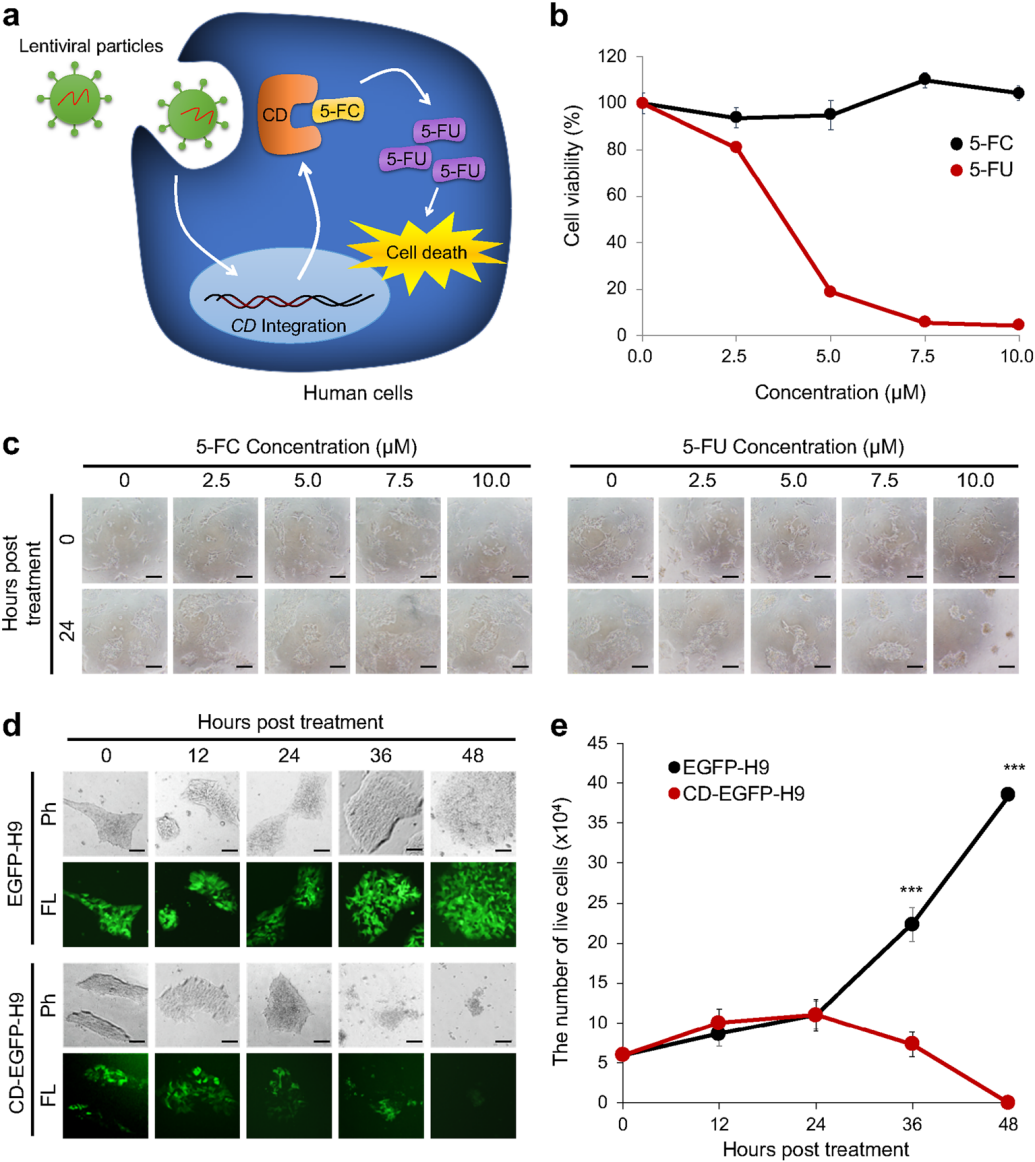
Removal of CD gene-integrated human ESCs by 5-FC treatment. **a** A schematic of the cytotoxic effects of 5-FC treatment on CD-integrated cells is shown. Genomic integration was mediated by lentiviral transduction. **b** The viability of H9 human ESCs treated with varying concentrations of 5-FC and 5-FU was measured using a WST-1 assay. All values are normalized to controls (0 µM). This experiment was performed in triplicate. **c** Representative images of the morphology of H9 cells treated with 5-FC or 5-FU were randomly acquired at the indicated time points. Scale bars represent 50 µm. **d** Phase contrast (Ph) and fluorescence (FL) images of EGFP-H9 and CD-EGFP-H9 cells were obtained every 12 h after treatment with 50 µM 5-FC. Scale bars represent 50 µm. **e** The number of live cells was counted every 12 h by trypan blue staining. This experiment was performed in triplicate. *** Represents *P* < 0.001 using Student’s *t*-test

Suicide genes are used to kill unwanted cells by converting pro-drugs to toxic drugs. Thus, we hypothesized that suicide gene-integrated cells would be negatively selected by the addition of a paired pro-drug (Fig. 1a). Previously, suicide genes have been used to remove pluripotent stem cells (PSCs) from differentiated cells because PSCs can form teratogenic tumors when transplanted^26^. However, suicide genes have not been used to select EF-iPSCs.

Among several suicide genes, we chose the CD gene because this gene has been used effectively and safely in gene therapy to eliminate tumor cells^22–25,33,34^. Furthermore, 5-FC conversion does not naturally occur in human cells^35^. There are *Escherichia coli* CD (bCD) and a *Saccharomyces cerevisiae* CD (yCD) genes. Because yCD has a 22-fold lower Km value for the conversion of 5-FC to toxic 5-FU than bCD, yCD more rapidly induces cytotoxicity^36^. Thus, we used yCD to achieve the rapid selection of EF-reprogrammed cells in this study.

### Negative selection of CD-integrated human embryonic stem cells

We generated EGFP- and CD-EGFP-integrated 293T cells using lentiviral vector transduction and treatment with 5-FC to confirm the negative selection of human cells that acquired genomic integration of the CD gene. Depending on the 5-FC concentration, the number of CD-EGFP-293T cells was significantly decreased (Supplementary Fig. 1a, b), and we found that the surviving CD-EGFP-transduced cells did not express EGFP (Supplementary Fig. 1a). In contrast, the survival and growth of EGFP-293T cells without the CD gene were not affected by 5-FC treatment (Supplementary Fig. 1a, b). Based on these data, we confirmed that the CD/5-FC combination works well in human cells.

Next, we sought to analyze the effectiveness of 5-FC or 5-FU treatment in human ESCs. The viability of human ESCs was not altered by treatment with up to 100 µM 5-FC (data not shown), whereas treatment with 2.5 µM 5-FU induced massive cell death (Fig. 1b). Notably, cells cultured with 5-FC showed normal human ESC morphology without spontaneous differentiation (Fig. 1c). Thus, we found that 5-FC treatment did not induce the death or differentiation of human ESCs. Then, we lentivirally transduced H9 human embryonic stem cells (ESCs) with EGFP or CD-EGFP under the control of the elongation factor 1-alpha (EF1-alpha) promoter to mimic the condition in which the CD gene is integrated into the iPSC genome. Virus-infected cells expressed EGFP and proliferated normally. We applied 50 µM 5-FC to EGFP-H9 or CD-EGFP-H9 cells. Similar to that observed in the 293T cells, 36 h of treatment with 50 µM 5-FC was sufficient to induce the death of CD-EGFP-H9 cells but not EGFP-H9 cells (Fig. 1d, e). Therefore, we confirmed that CD-integrated human pluripotent stem cells (PSCs) can be negatively selected by 5-FC treatment and that 5-FC treatment alone is not toxic.

### Rapid isolation of EF-iPSCs by the combined use of CD/5-FC

**Fig. 2:**
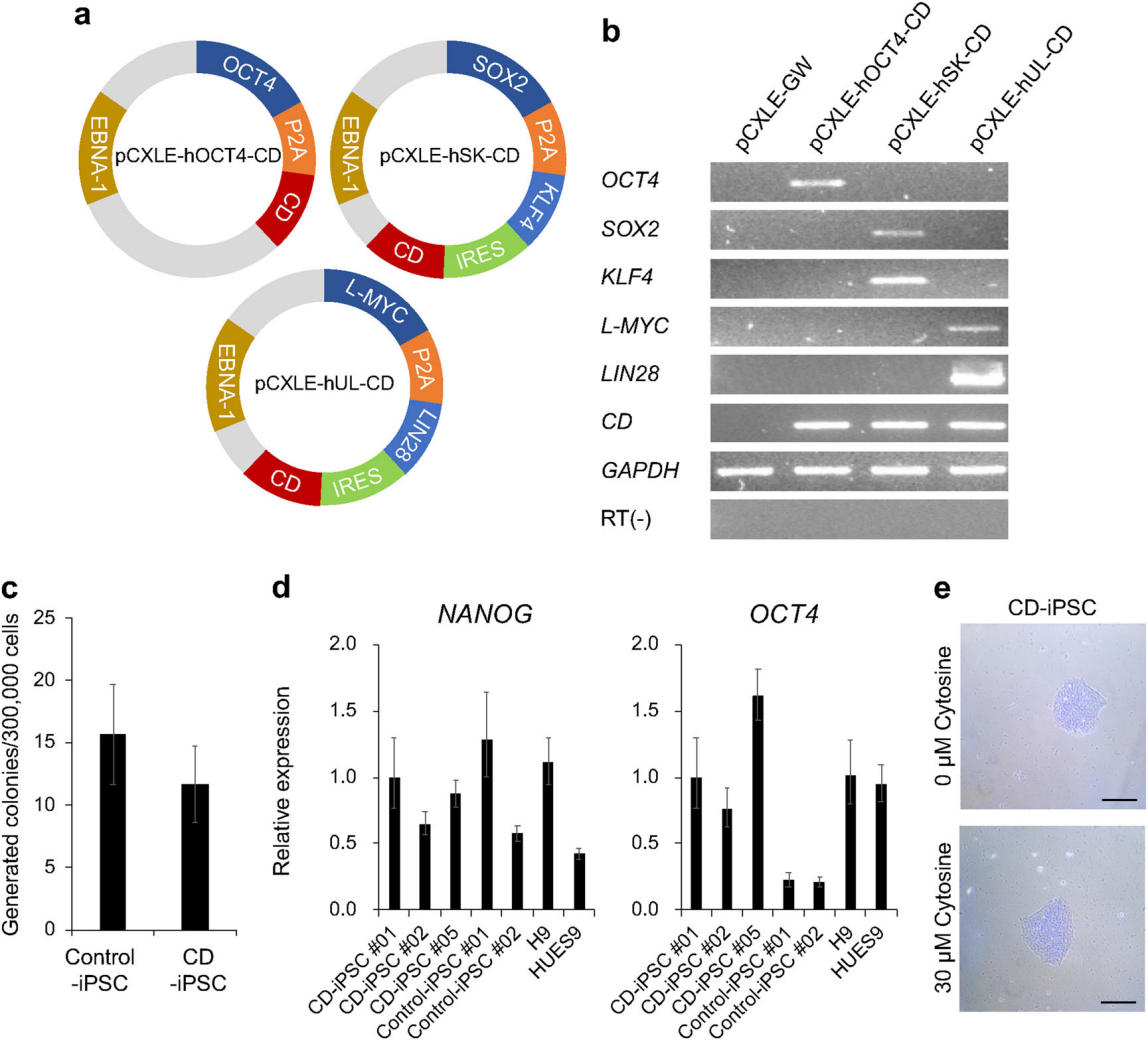
iPSC reprogramming with CD episomal vectors. **a** Schematics of the three CD episomal vectors are shown. **b** RT-PCR was conducted to confirm the expression of exogenes in each CD vector in 293T cells. The pCXLE-GW plasmid is an empty vector that was used as a negative control. **c** The number of generated colonies was counted. This experiment was performed in triplicate. **d** Quantitative PCR was conducted to evaluate the expression levels of *OCT4* and *NANOG* in CD-iPSCs, control-iPSCs, and ESCs (H9 and HUES9). **e** Representative images of CD-iPSCs treated with or without cytosine

According to Schlaeger et al., 33.3% of newly isolated iPSC clones are accompanied by episomal vectors until passage 12^15^. Indeed, when we generated iPSCs using conventional episomal vectors, approximately 13 passages were required to eliminate the episomal vectors. After we verified the usability of the CD/5-FC combination for removing PSCs, we constructed episomal vectors harboring the CD gene (CD episomal vectors). The CD gene was linked to other genes through a P2A or IRES sequence (Fig. 2a), and we verified the expression of CD episomal vectors in 293T cells (Fig. 2b). Next, we generated iPSCs using CD episomal vectors and compared them to conventional episomal vectors. The reprogramming efficiency of CD episomal vectors was not significantly different from that of control vectors (Fig. 2c). Moreover, we observed similar expression levels for *OCT4* and *NANOG* transcripts in ESCs and iPSCs generated by two different vector systems (Fig. 2d). Because CD may be able to deplete cytosine, we added cytosine to the media. However, regardless of the addition of cytosine, the iPSCs generated using CD episomal vectors (CD-iPSCs) exhibited intact morphology without any differentiation (Fig. 2e).

**Fig. 3:**
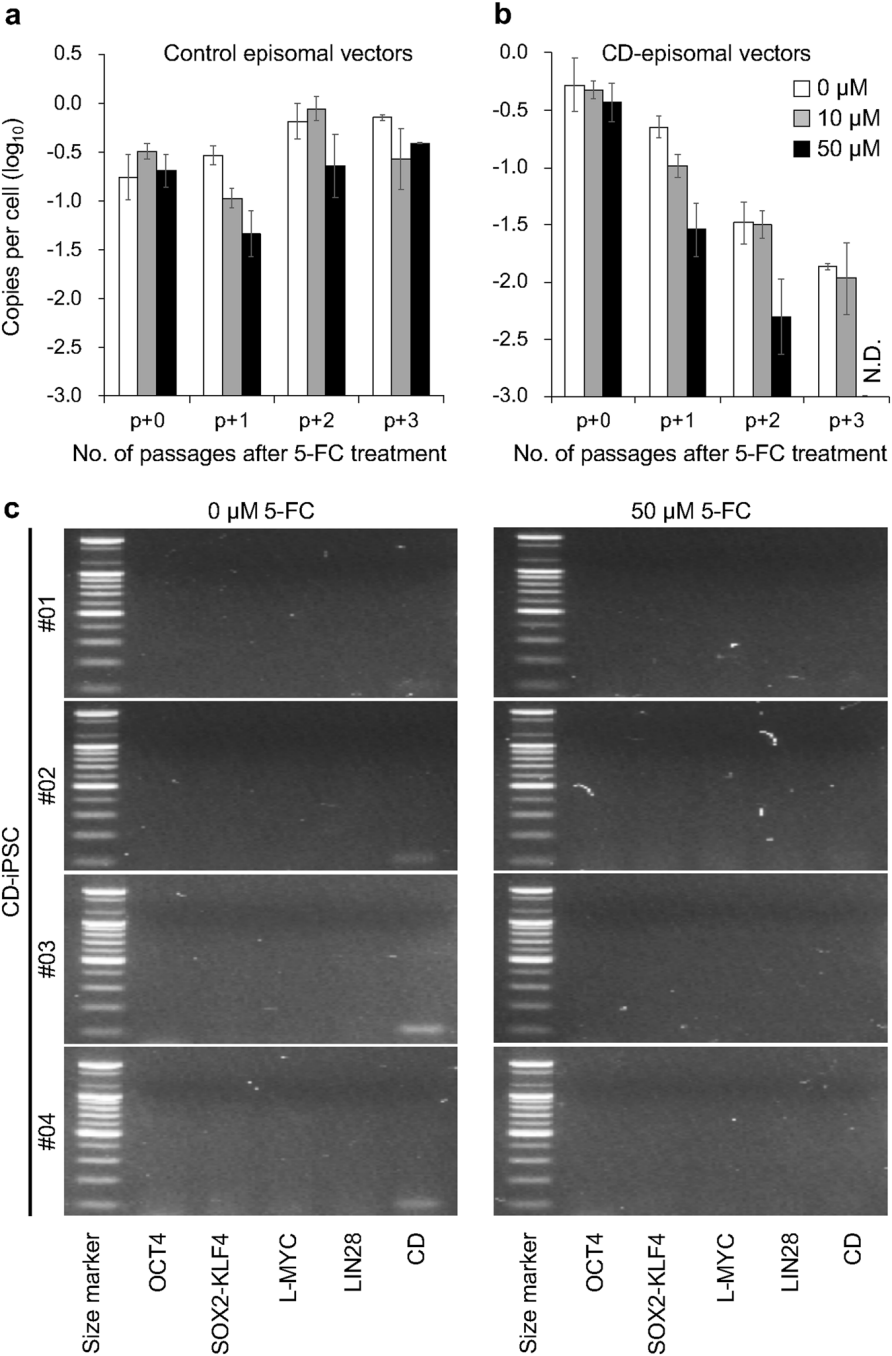
Rapid elimination of residual CD episomal vectors in human iPSCs. A qPCR analysis was used to measure the copy numbers of the remaining episomal vectors in control-iPSCs (**a**) and CD-iPSCs (**b**) during serial culture with or without 5-FC. N.D. indicates not detected. In these experiments, the control- and CD-iPSC samples used were mixtures of nine independent iPSC colonies. **c** A PCR analysis was used to detect the exogenously introduced reprogramming factors and the CD gene. Total DNA from CD-iPSC #01, #02, #03, and #04 with or without 5-FC treatment was used as a template. A 100 bp size marker ladder (Bioneer) was used

After the iPSC colonies were obtained, we added 5-FC to the iPSC culture media. In this experiment, we intentionally mixed nine different iPSC colonies to reduce the influence of variations in individual cells from different colonies. To confirm the episomal vector copy number, we used quantitative PCR of the Epstein-Barr nuclear antigen-1 (*EBNA-1*) sequence, which plays an important role in episomal vector replication. Surprisingly, we observed a rapid reduction in the number of episomal vectors in the CD-iPSCs when we treated them with 50 µM 5-FC and complete removal after four passages. Notably, iPSCs generated using conventional vectors contained a high number of episomal vectors, even after four passages (Fig. 3a, b, and Table 1). In addition, we did not observe any signs of growth defects or differentiation in control- and CD-iPSCs after 5-FC treatment, indicating that 5-FC treatment did not influence the maintenance of iPSCs. Based on these results, we propose that the combined use of CD/5-FC rapidly removes residual episomal vectors after iPSC reprogramming.

**Table 1:** Comparison of the effects of 5-FC treatment on the time required to eliminate the CD episomal vectors

Cell line	5-FC (μM)	Minimum number of passages needed for EF-cell generation
Control-iPSC #01^a^	0	>6
	50	>6
CD-iPSC #01^a^	0	>6
	50	4
CD-iPSC #02^b^	0	>1
	50	1
CD-iPSC #03^b^	0	>2
	50	2
CD-iPSC #04^b^	0	>2
	50	2
CD-iNSC^b^	0	>3
	50	1

^a^This sample is a mixture of nine independent iPSC colonies

^b^For this experiment, we dissociated the colonies into single cells

Compared to the amount of time required to remove residual episomal vectors using conventional vectors, taking more than 70 days, our method only takes 20–30 days and four passages. This improvement not only reduces the time needed but also reduces the effort and cost of maintaining many individual iPSC clones, which need to be screened and selected to obtain EF-iPSCs. Thus, we sought to further shorten the period needed to isolate EF-iPSCs. We dissociated the isolated CD-iPSC colonies into single cells and replated them with 50 µM 5-FC. Surprisingly, residual vectors were not detectable after only one or two passages (Table 1).

In a previous study, it was shown that the partial integration of reprogramming factors occurred occasionally in episomal vector-generated iPSCs^2^. To detect any integration of exogenously introduced genes, we performed PCR for the region that spanned the exogenously introduced reprogramming factors in CD-iPSCs. We confirmed that the PCR primers used in this experiment annealed with the CD episomal vectors but not with genomic DNA (Supplementary Fig. 2). Consistent with the data from the detection of residual EBNA-1 DNA (Fig. 3a, b), we found a complete loss of exogenes in CD-iPSCs after 50 µM 5-FC treatment (Fig. 3c). Overall, these results demonstrated that the combined use of CD/5-FC rapidly eliminated residual CD episomal vectors.

**Fig. 4:**
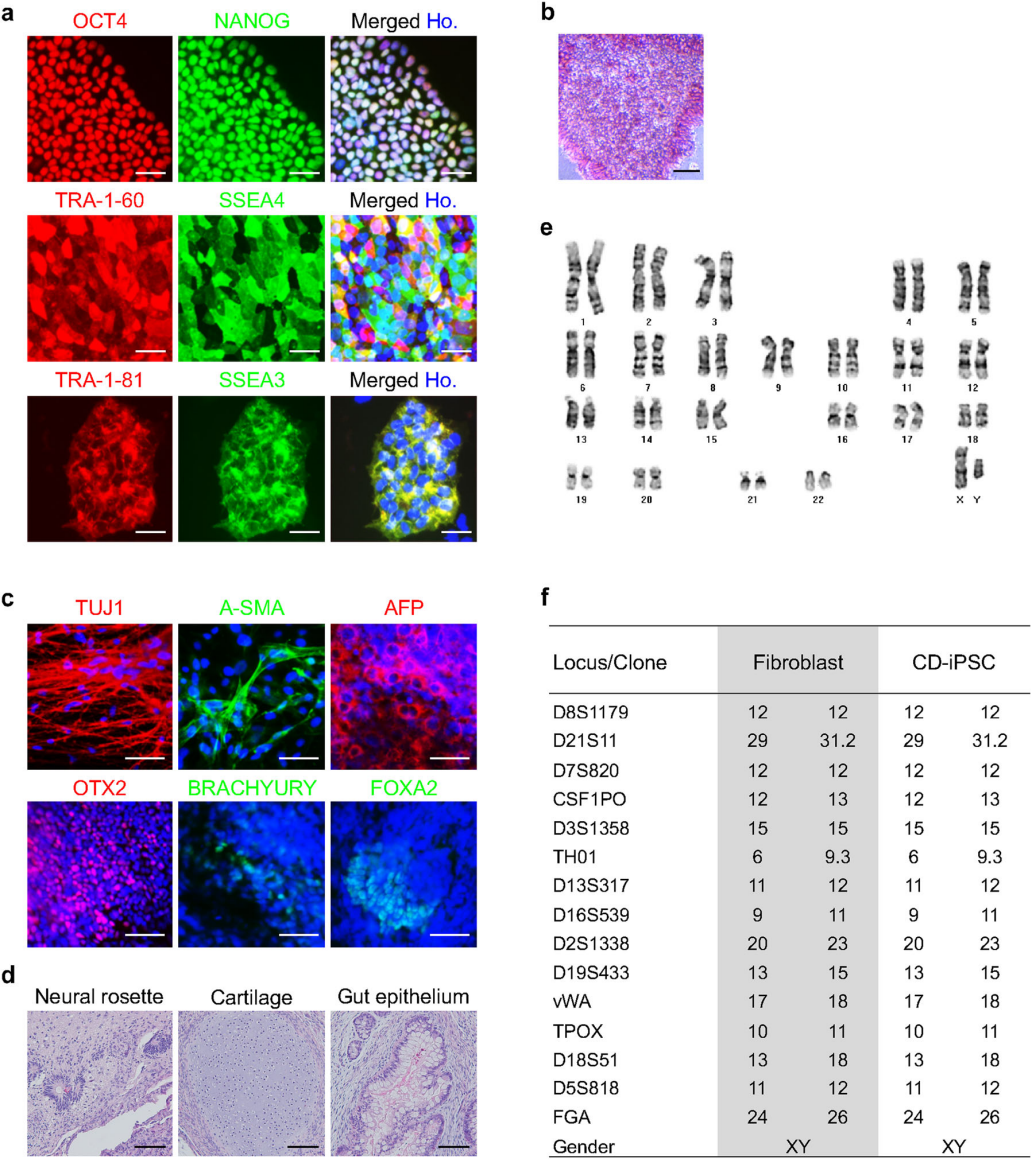
Characterization of the selected EF-iPSCs treated with the CD/5-FC combination. The expression of various pluripotency markers was confirmed by immunocytochemistry (**a**) and AP staining (**b**). Scale bars represent 50 µm and 100 µm, respectively. **c** The in vitro differentiation potential was confirmed by immunocytochemistry of several markers in the three germ layers. The iPSCs were differentiated into EBs. Hoechst 33342 was used to stain the nuclei (blue). Scale bars represent 50 µm. **d** A teratoma formation assay was performed in immunodeficient mice to confirm the in vivo differentiation potential. The resulting teratomas were stained with H&E. Scale bars represent 100 µm. **e** G-banding analysis confirmed that the selected iPSCs had normal human karyotypes. **f** STR analysis confirmed that the CD-iPSCs were derived from the original CRL2097 fibroblasts. Ho. represents Hoechst 33342

The isolated EF-iPSCs showed high expression of pluripotency markers, such as OCT4, NANOG, TRA-1-60, TRA-1-81, SSEA3, SSEA4, and AP (Fig. 4a, b), and underwent differentiation into three germ layers in vitro and typical teratoma formation in vivo (Fig. 4c, d). The cultured EF-iPSCs had a normal karyotype, as confirmed by a G-banding analysis (Fig. 4e), and showed the same STR analysis result as CRL2097 fibroblasts, confirming the origin of the EF-iPSCs (Fig. 4f). We showed that EF-iPSCs maintained the expression of pluripotency markers over 20 passages in culture (Supplementary Fig. 3). Thus, we concluded that the combined use of CD/5-FC enabled the rapid isolation of EF-iPSCs as early as passage 1, and the isolated EF-iPSCs showed the characteristics of normal human PSCs.

### Direct reprogramming to neural stem cells using CD vectors

**Fig. 5:**
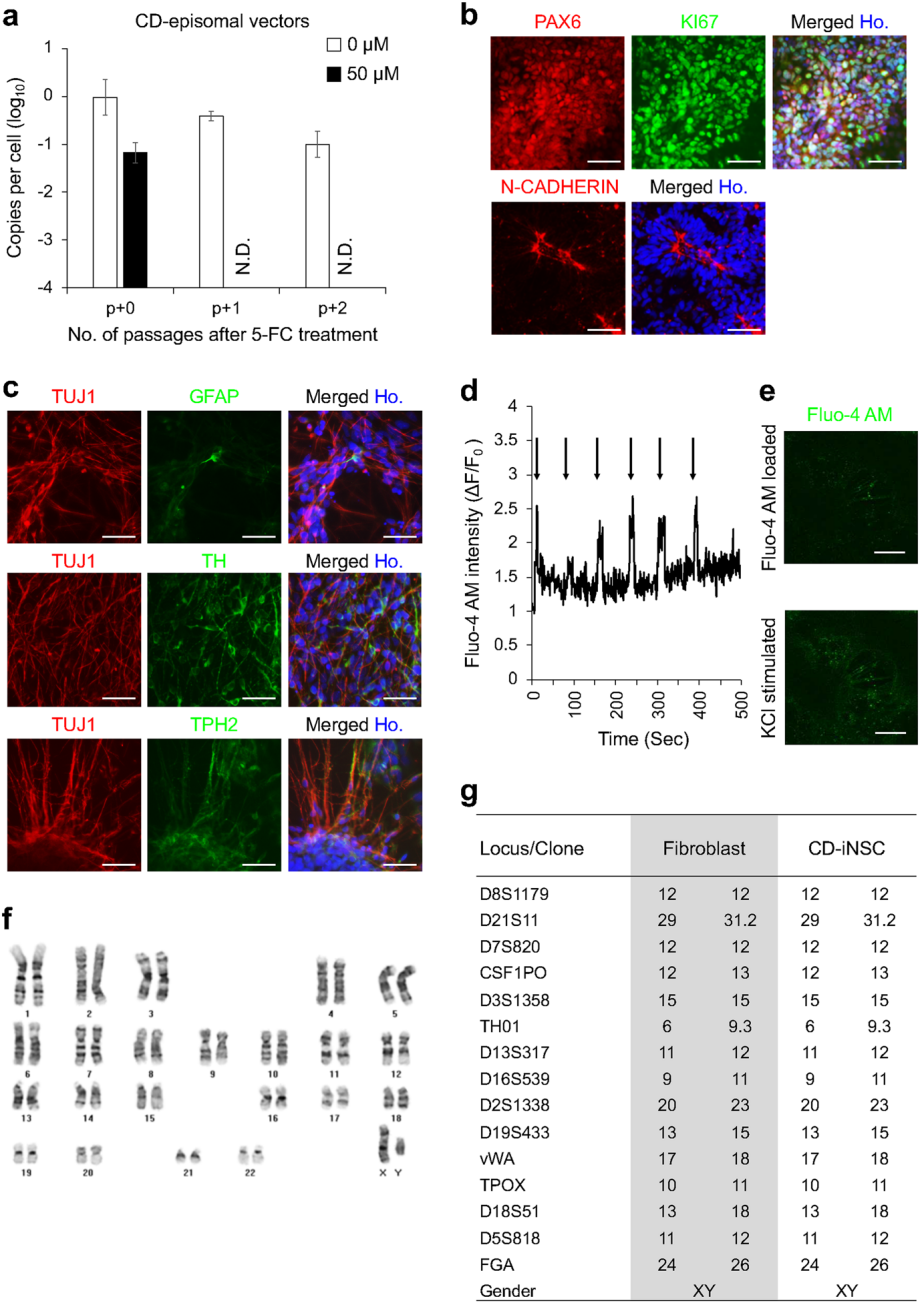
Rapid elimination of residual CD episomal vectors in human-induced neural stem cells. **a** qPCR analysis of total DNA in CD-iNSCs cultured with or without 5-FC was used to measure the copy numbers of residual CD episomal vectors. N.D. indicates not detected. **b** Immunocytochemistry of NSC markers that were expressed in the isolated EF-iNSCs. Scale bars represent 100 µm. **c** Images of immunocytochemical staining of neuronal and glial markers are shown to confirm the differentiation potential of isolated EF-iNSCs. **d** KCl-induced transient Ca^2+^ (real-time) in neurons at eight weeks post differentiation. The arrow represents KCl stimulation. **e** Representative images of Fluo-4 AM-loaded neurons pre and post KCl administration. Scale bars represent 200 µm. **f** G-banding analysis of EF-iNSCs showed normal human karyotypes. **g** STR analysis revealed that the CD-iNSCs were derived from the original CRL2097 fibroblasts

Because we and other researchers have shown that pluripotency reprogramming factors, such as OCT4, SOX2, KLF4, and C-MYC, can also be used for the direct reprogramming of somatic cells into neural stem/progenitor cells^28,29,37–42^, we attempted to use CD episomal vectors for the direct reprogramming of human fibroblasts into induced neural stem cells (iNSCs). We introduced our CD episomal vectors into human fibroblasts and reprogrammed them as was previously reported^28^. As expected, we observed small colonies that were morphologically identical to iNSCs. After the isolation of the emerging iNSC colonies, the colonies were treated with 50 µM 5-FC, as was done during the EF-iPSC reprogramming. Surprisingly, we could isolate EF-iNSCs that did not contain any episomal vectors after only one passage (Fig. 5a). In contrast, iNSCs without 5-FC treatment still contained CD episomal vectors after continuous passaging (Fig. 5a). We confirmed that the isolated EF-iNSCs showed the expression of the required NSC markers, such as paired box protein 6 (PAX6), N-CADHERIN, and KI67 (Fig. 5b). We found typical neural rosette formation by examining the luminal expression of N-CADHERIN (Fig. 5b). Upon further spontaneous differentiation, EF-iNSCs efficiently produced neuron-specific class III β-tubulin (TUJ1)-positive neurons and glial fibrillary acidic protein (GFAP)-positive glial cells (Fig. 5c). We also found that EF-iNSCs can differentiate into various subtypes of neurons, including tyrosine hydroxylase (TH)-positive dopaminergic and tryptophan hydroxylase 2 (TPH2)-positive serotonergic neurons (Fig. 5c). To assess whether EF-iNSC-derived neurons have functional properties, we confirmed the occurrence of calcium influx under KCl-induced depolarized conditions. We observed transient calcium amplitudes (F/F0 based on the calcium indicator Fluo-4 AM), indicating the functionality of EF-iNSC-derived neurons in vitro (Fig. 5d, e). In addition, we examined marker expression for mature neuron and neurotransmitter receptor (Supplementary Fig. 4). We found that the expression of every marker we tested, including microtubule-associated protein 2 (MAP2), neuronal nuclei antigen (NEUN), synapsin1, gamma-aminobutyric acid B receptor 1 (GABBR1, GABA receptor), glutamate receptor subunit zeta 1 (GRIN1, NMDA receptor), and glutamate receptor 2 (GRIA2, AMPA receptor) was increased in differentiated neurons compared with EF-iNSCs. We confirmed that the EF-iNSCs possessed a normal karyotype (Fig. 5f), and the STR analysis indicated that the EF-iNSCs were derived from CRL2097 fibroblasts (Fig. 5g). Based on these data, we propose that we could expand the applicability of our CD episomal vectors to facilitate the direct reprogramming and isolation of EF-iNSCs rapidly within a single passage.

## Discussion

We developed a new method for the rapid isolation of EF-iPSCs and EF-iNSCs that has not yet been reported that uses episomal vectors containing the CD gene. Although several nonintegrating reprogramming systems have been developed^4–7,9,11^, the removal of the residual vectors is usually overlooked and not regarded as critical. However, when producing high quality reprogrammed cells, the amount of effort and time required to isolate EF-reprogrammed cells is not negligible. Previously, Du et al. reported a method for the negative selection of porcine EF-iPSCs that used thymidine kinase, but it could not remove exogenes completely^43^. In this study, we showed that EF-reprogrammed cells could be easily isolated when our suicide gene-embodied episomal vectors were used. In addition, PSCs with an integrated copy of the suicide gene were rapidly removed. The change is simple, but the effect is considerable. We successfully obtained EF-iPSCs and EF-iNSCs within one week with the combined use of CD/5-FC (Table 1).

However, the underlying mechanism involved in the rapid generation of EF-reprogrammed cells is not clear. There are two possible mechanisms involved in the combined use of CD/5-FC: the killing of cells harboring the suicide gene in episomal vectors and the elimination of episomal vectors from cells. The CD/5-FC combination can kill cells and their neighbors by the bystander effect^21^. However, we did not observe massive cell death under 5-FC treatment. Thus, we assume that CD episomal vectors could be an unnecessary or hazardous burden for cells when 5-FC is present, possibly causing the cells to not carry the episomal vectors during cell division. Similarly, it is well known that *S. cerevisiae* containing *scURA3*, which is a suicide gene, removes this gene by itself under prodrug treatment conditions^44,45^. We assume that this elimination process also lowers the chances of genomic integration by reducing the time that episomal vectors are inside cells.

As shown in our results, EF-iPSCs and EF-iNSCs that were generated with our CD episomal vectors showed normal characteristics and karyotypes (Figs. 4, 5). These results indicate that the CD/5-FC combination does not affect their original properties. Previous studies also show the safety of the CD/5-FC combination for clinical applications^22–25^. For tumor-specific suicide gene activation, the intratumoral injection of vectors with tumor-specific genes, such as erbB-2, which is a promoter in breast cancer, is used. The authors of the study showed that there were no significant side effects and no dose-dependent toxicity, demonstrating the safety of the suicide gene approach. Because 5-FU results in the inhibition of DNA synthesis, treatment with CD/5-FC over the long-term and at high concentrations may raise safety concerns. However, the concentration of 5-FC we used was nominal, and the CD vectors were removed from the cells rapidly within one week in vitro. Compared to methods used in previous studies, we assume that our methods cannot trigger dose-dependent toxicity and that the cells generated by our method are intact. Thus, the CD/5-FC combination is suitable for reprogramming and clinical applications.

Exogene expression can hinder and change the function of intact cells^3^. Especially in iPSCs, differentiation into a certain cell type could be interrupted by the residual expression of the reprogramming factors. When such expression is maintained in differentiated cells, this may cause dedifferentiation or tumorigenicity^46^. Thus, EF cells are mandatory for precise research and safe clinical application. Because our new reprogramming method can be used to obtain intact EF-reprogrammed cells simply and efficiently, we expect that our system will lower the cost and time required for the generation and application of reprogrammed cells.

After we confirmed the usability of the CD/5-FC system for generating EF-iPSCs, we applied it and confirmed its use in direct reprogramming or transdifferentiation. Because we previously demonstrated a novel paradigm for transdifferentiation in which pluripotent reprogramming factors are used but the iPSC stage is skipped in mouse and human cells^28,29^, we were able to directly use our CD episomal vectors to generate induced neural stem cells (iNSCs) by direct reprogramming. Considering the advantages of direct reprogramming, including the reduced probability of tumorigenicity caused by pluripotent cells and the fast generation of target cells by skipping the pluripotent stage, our new episomal vector system is also appropriate for applications to direct reprogramming. Although we only showed the use of the CD/5-FC combination in isolating EF cells after establishing iNSCs, we expect to be able to control the expression of reprogramming factors by treatment with 5-FC during direct reprogramming. This may help to further optimize and increase the understanding of the process of direct reprogramming in human cells.

## Supplementary information

Supplementary Figure 1

Supplementary Figure 2

Supplementary Figure 3

Supplementary Figure 4

Supplementary Table 1

Supplementary Table 2
