# Supplementary material for: Efficient exogenous DNA-free reprogramming with suicide gene vectors
Source: Exp Mol Med. 2019 Jul 19;51(7):82. doi: 10.1038/s12276-019-0282-7 (PMC6802735; doi:10.1038/s12276-019-0282-7)
Supplement: Supplementary file 1 — Supplementary Figure 1 [file 12276_2019_282_MOESM1_ESM.docx]

**Supplementary information Figure 1**


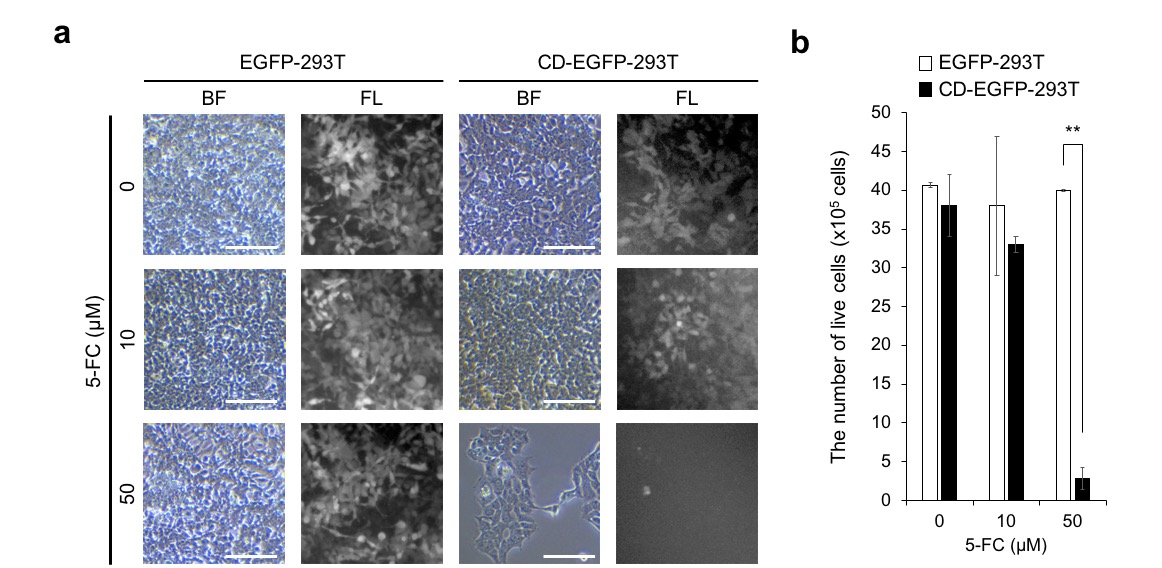


**Supplementary Figure 1.** Removal of *CD* gene-integrated 293T cells by the 5-FC treatment. (a) Representative bright field (BF) and fluorescence (FL) images of EGFP-293T or CD-EGFP-293T cells were acquired on day six after the 5-FC treatment. Scale bars represent 50 µm. (b) The number of live cells was counted on day six after 5-FC treatment. Trypan blue staining was used to count the live cells. **P*<0.05; ***P*<0.01 using Student’s *t*-test.
