# Supplementary material for: Efficient exogenous DNA-free reprogramming with suicide gene vectors
Source: Exp Mol Med. 2019 Jul 19;51(7):82. doi: 10.1038/s12276-019-0282-7 (PMC6802735; doi:10.1038/s12276-019-0282-7)
Supplement: Supplementary file 2 — Supplementary Figure 2 [file 12276_2019_282_MOESM2_ESM.docx]

**Supplementary information Figure 2**

**Supplementary Figure 2.** A PCR analysis was used to detect the each exogenously introduced reprogramming factors and CD. Genomic DNA was used as a negative control and a mixture of three CD-episomal vectors was used as a positive control.
