# Supplementary material for: Efficient exogenous DNA-free reprogramming with suicide gene vectors
Source: Exp Mol Med. 2019 Jul 19;51(7):82. doi: 10.1038/s12276-019-0282-7 (PMC6802735; doi:10.1038/s12276-019-0282-7)
Supplement: Supplementary file 3 — Supplementary Figure 3 [file 12276_2019_282_MOESM3_ESM.docx]

**Supplementary information Figure 3**

**Supplementary Figure 3.** Characterization of the long-term cultured EF-iPSCs. The expression of OCT4, NANOG, TRA-1-60, SSEA4, TRA-1-81, and SSEA3 was confirmed by immunocytochemistry. Hoechst33342 was used to stain the nuclei (blue). Scale bars represent 50 µm.
