# Supplementary material for: Efficient exogenous DNA-free reprogramming with suicide gene vectors
Source: Exp Mol Med. 2019 Jul 19;51(7):82. doi: 10.1038/s12276-019-0282-7 (PMC6802735; doi:10.1038/s12276-019-0282-7)
Supplement: Supplementary file 4 — Supplementary Figure 4 [file 12276_2019_282_MOESM4_ESM.docx]

**Supplementary information Figure 4**

**Supplementary Figure 4.** Confirmation of the mRNA expression levels of neuronal and neurotransmitter receptor genes. Quantitative PCR was conducted to evaluate the expression level of *MAP2*, *NEUN*, *SYNAPSIN1*, *GABBR1*, *GRIN1*, and *GRIA2* in CD-iNSCs and their differentiated progenies.
