# Supplementary material for: Efficient exogenous DNA-free reprogramming with suicide gene vectors
Source: Exp Mol Med. 2019 Jul 19;51(7):82. doi: 10.1038/s12276-019-0282-7 (PMC6802735; doi:10.1038/s12276-019-0282-7)
Supplement: Supplementary file 5 — Supplementary Table 1 [file 12276_2019_282_MOESM5_ESM.pdf]

Supplementary Table 1. List of the primers used in this study.

Gene	Primer (Forward)	Primer (Reverse)
FBXO15	GCCAGGAGGTCTTCGCTGTA	AATGCACGGCTAGGGTCAAA
EBNA1	AGACACATCTGGACCAGAAGGCTC	TGTTCCACCGTGGGTCCCTTTG
OCT4	GAGGAGTCCCAGGACATCAA	AATAGAACCCCCAGGGTGAG
SOX2	GCCGAGTGGAAACTTTTGTCG	GGCAGCGTGTACTTATCCTTCT
KLF4	CCCACATGAAGCGACTTCCC	CAGGTCCAGGAGATCGTTGAA
L-MYC	GTCATCGCCCCGTATCTCC	TGGCAAGTCGATATACCTCCA
LIN28A	TGCGGGCATCTGTAAGTGG	CCTCTCGAAAGTAGGTTGGCT
CD	GTGACAGGGGGAATGGCAAGCA	GCATGGAGACAGCGTCGTATACAA
NANOG	AACGTTCTGCTGGACTGAGC	ATGCTTCAAAAGCAAGGCAAG
MAP2	CGAAGCGCCAATGGATTCC	TGAACTATCCTTGCAGACACCT
NEUN	CCCATCCCGACTTACGGAG	GCTGAGCGTATCTGTAGGCT
SYNAPSIN1	AGTTCTTCGGAATGGGGTGAA	CAAACTGCGGTAGTCTCCGTT
GABBR1	AGCCGTCATTACAAGATGAACTT	TGGTCTCAGGCGATTGTCATAA
GRIN1	ACCCCAAGATCGTCAACATTG	GGCTAACTAGGATGGCGTAGA
GRIA2	CACCCCACATCGACAATTTGG	GACGTGGAGTGTTCCGCAA
Exo-OCT4	TGCTGGGTCTCCTTTCTCAGGG	CATTGGCCCGGGATTCTCTTC
Exo-SOX2-KLF4	CGCCCACCTACAGCATGTCCTA	GCTTCATGTGGGAGAGCTCCTC
Exo-L-MYC	TGCCTTCTTCTTTTTCCTACAGCTC	TTTCTTCCAGATGTCCTCGCTG
Exo-LIN28A	TGCGGGCATCTGTAAGTGG	GGAACCCTTCCATGTGCAG
Exo-CD	TGACAGGGGGAATGGCAAGC	ACATCCGCCAATAGGAACACCAC
