# Supplementary material for: Efficient exogenous DNA-free reprogramming with suicide gene vectors
Source: Exp Mol Med. 2019 Jul 19;51(7):82. doi: 10.1038/s12276-019-0282-7 (PMC6802735; doi:10.1038/s12276-019-0282-7)
Supplement: Supplementary file 6 — Supplementary Table 2 [file 12276_2019_282_MOESM6_ESM.pdf]

Supplementary Table 2. List of antibodies used in this study

Antibody	Dilution	Supplier (Catalog Number)
Rabbit anti-OTX2	1:100	Abcam (#ab21990)
Mouse anti-TUJ1	1:5000	BioLegend (#801201)
Mouse anti-αSMA	1:200	Sigma (#A5228)
Goat anti-FOXA2	1:100	R&D Systems (#AF2400)
Rabbit anti-AFP	1:400	Dako (#A0008)
Goat anti-BRACHYURY	1:300	SantaCruz (#sc-17745)
Rabbit anti-OCT4	1:500	SantaCruz (#sc-9081)
Goat anti-NANOG	1:70	R&D Systems (#AF1997)
Mouse anti-TRA-1-81 (IgM)	1:500	Millipore (#MAB4381)
Mouse anti-TRA-1-60 (IgM)	1:500	Millipore (#MAB4360)
Rat anti-SSEA3 (IgM)	1:500	Millipore (#MAB4303)
Mouse anti-SSEA4	1:500	Millipore (#MAB4304)
Rabbit anti-GFAP	1:500	Dako (#Z0334)
Rabbit anti-TH	1:1000	Millipore (#AB152)
Rabbit anti-TPH2	1:2000	Novus Biologicals (#100-74555)
Rabbit anti-PAX6	1:500	BioLegend (#901301)
Mouse anti-KI67	1:500	BD (#556003)
Rabbit anti-N-CADHERIN	1:200	SantaCruz (#sc-7939)
